# Carbolic Acid

**Published:** 1877-01

**Authors:** 


### Miscellaneous.

### CARBOLIC ACID.

It would be safe to assert that one can not take up a medical journal nowadays without finding something new about carbolic acid. Brought into notice at first for its antiseptic properties, it has found innumerable other important applications in therapeutics, and the list is ever increasing. Professor J. S. Wright, in a recent lecture remarks:—

Antiseptic surgery has become famous, so has carbolic acid; and the fame of carbolic acid comes from its antiseptic power. But carbolic acid has other properties; carbolic acid is an astringent, arresting bleeding from small vessels, and preventing the entrance of septic germs into the lymph spaces, lymphatics, and blood capillaries; it is a local anodyne, alleviating the pain and the shock of injury; it is a local anæsthetic, mitigating and preventing the pain and shock of operations; it slows, or inhibits the amœboid motion of the white cells, often modifying in a favorable manner the process of plastic infiltration; it diminishes reflex irritation, controlling the action of the vaso-motor nerves, limiting congestion and exudation, and tending to subdue inflammation. See how many substantial reasons why carbolic acid may be used in surgery; and these reasons are not because carbolic acid is antiseptic.

Carbolated camphor is a preparation which is gaining favor in foreign medical circles. Dr. Soulez, in the Bulletin de Therapeutique gives the following account of this compound:—

It is made by dissolving 2.5 grammes of powdered camphor in one gramme of carbolic acid (a solution of the strength of nine grammes in one gramme of alcohol). The The so'ution is of an oleaginous consistency, pale yellow, smelling slightly of camphor, but having none of the disagreeable odor of carbolic acid. It boils at a slightly elevated tempature without decomposing, and also by the addition of concentrated alcohol, which throws down the camphor in crystals; similarly, if a boiling solution of carbolated camphor is poured into cold water it instantly solidifies. It is miscible in all proportions with olive and almond oils. Chemical examination shows that the carbolic acid and camphor are not altered, and that they preserve all their properties in the solution.

Dr. Soulez claims as the result of using this as a dressing: 1. Diminution of reaction after severe operations; 2. Cessation or amelioration of pain; 3. Less abundant suppuration.

We need hardly say that in all medicinal applications of carbolic acid, the purity of the agent is of the utmost importance. The acid exhibited at Philadelphia by Messrs. Billings, Clapp and Co.; of Boston, attracted much attention by its superior quality. Physicians and druggists who wish a standard and uniformly reliable article will be sure of getting it if it comes from that house.

